# Exploring Co-Residential Grandparents’ Time Investments in Grandchildren in the United States

**DOI:** 10.1093/geroni/igaf122.4114

**Published:** 2025-12-31

**Authors:** Abigail Stephan, Sarah Flood, Jill Juris

**Affiliations:** Clemson University, Clemson, South Carolina, United States; University of Minnesota, Minneapolis, Minnesota, United States; Appalachian State University, Boone, North Carolina, United States

## Abstract

More than six million households across the United States contain both grandparents and grandchildren. Despite the prevalence of co-residential grandparents in skipped-generation grandfamilies (containing grandchildren and grandparents but no parent generation) or multigenerational households (containing children, parents, and grandparents), detailed knowledge of grandparents’ time-based contributions to grandchildren’s development is currently limited. Mirroring the vast parental time investment literature, we use nationally representative time diary data from the American Time Use Survey (2003-2022) to test three hypotheses–linked lives, gender, and grandchild needs–around the amount of time grandparents (N = 5,557) spend with grandchildren under age 18. Bivariate results show that (1) co-residential grandparents spend an average of 3.25 hours per day with their grandchildren, (2) co-residential grandmothers spend more time caring for grandchildren than co-residential grandfathers, (3) grandparents in grandfamilies spend more time with grandchildren than grandparents in multigenerational households, and (4) grandparent time investments decline with grandchild age. Analyzing the composition of co-residential grandparents’ time with grandchildren and the intersections between gender, family structure, and grandchild needs reveals a more complex and nuanced picture of grandparent investments in grandchildren. Results highlight the need for policies and support systems tailored to co-residential grandparents with varying circumstances who invest in their grandchildren’s care and development.

